# Higher Serum Brain-Derived Neurotrophic Factor Levels Are Associated With a Lower Risk of Cognitive Decline: A 2-Year Follow Up Study in Community-Dwelling Older Adults

**DOI:** 10.3389/fnbeh.2021.641608

**Published:** 2021-06-22

**Authors:** Yoshinori Fujiwara, Kazushige Ihara, Mitsugu Hachisu, Hiroyuki Suzuki, Hisashi Kawai, Ryota Sakurai, Hirohiko Hirano, Paulo H. M. Chaves, Masahiro Hashizume, Shuichi Obuchi

**Affiliations:** ^1^Research Team for Social Participation and Community Health, Tokyo Metropolitan Institute of Gerontology, Tokyo, Japan; ^2^Department of Social Medicine, Hirosaki University Graduate School of Medicine, Hirosaki, Japan; ^3^Division of Clinical Pharmacy, Department of Pharmaceutical Therapeutics, Pharmacy School, Showa University, Tokyo, Japan; ^4^Research Team for Human Care, Tokyo Metropolitan Institute of Gerontology, Tokyo, Japan; ^5^Research Team for Promoting Independence and Mental Health, Tokyo Metropolitan Institute of Gerontology, Tokyo, Japan; ^6^Benjamin Leon Center for Geriatric Research and Education, Department of Translational Medicine, Herbert Wertheim College of Medicine, Florida International University, Miami, FL, United States; ^7^Department of Psychosomatic Medicine, School of Medicine, Toho University, Tokyo, Japan

**Keywords:** serum BDNF, cognitive decline, Montreal Cognitive Assessment-Japanese version, prospective observational study, community-dwelling older adults

## Abstract

**Objective:**

To assess the relationship of serum brain-derived neurotrophic factor (BDNF) levels with the subsequent short-term decline in cognitive functioning in community-dwelling older adults.

**Design:**

Two-year prospective, observational study.

**Setting and Participants:**

The study included 405 adults aged 65–84 years, initially free of a dementia diagnosis who were living in Tokyo, Japan.

**Methods:**

Participants underwent health assessments at baseline (2011) and follow-up (2013). Serum BDNF levels and scores from the Montreal Cognitive Assessment-Japanese version (MoCA-J) were systematically measured. Logistic regression was used to estimate the odds of cognitive decline between baseline and follow-up assessments in the full MoCA-J scale (operationally defined as a decrease of two or more points), as well as in MoCA-J subscales (decline of one or more points in a specific subscale), as a function of serum BDNF level, adjusting for baseline demographics, prevalent chronic diseases, and baseline cognitive scores.

**Results:**

Among individuals who performed worse on the full MoCA-J at baseline (i.e., scores in the bottom quartile [≤21], which is consistent with a mild cognitive impairment status), but not among those who performed better (top 3 quartiles), those with highest baseline serum BDNF levels (top quartile) had lower odds of subsequent decline in the full MoCA-J scale than those with lowest (bottom quartile); i.e., odds ratio (OR): 0.10 (95% confidence interval [CI]: 0.02–0.62; *p* = 0.013). Regarding MoCA-J subscales, adjusted odds of decline in the executive function subscale, but not in the other five subscales, were substantially low among those with highest baseline serum BDNF levels (top quartile), as compared to those with the lowest (bottom quartile), i.e., OR: 0.27 (95% CI:0.13–0.60; *p* < 0.001).

**Conclusion and Implications:**

Higher serum BDNF levels were associated with a lower risk of decline in cognitive function in a sample of community-dwelling older Japanese adults. Risk varied across cognitive subdomains and according to baseline cognition. This warrants further research to evaluate the added-value of serum BDNF in health promotion initiatives directed toward cognitive decline prevention in community-dwelling older adults.

## Introduction

As people age, their cognitive function declines more rapidly in areas of fluid intelligence, including executive function, processing speed, working memory, and reasoning (Park and Reuter-Lorenz, 2009). The prevention of such accelerated age-related cognitive decline, which threatens the quality of life of older adults, is a major area of public health and clinical research.

Brain deterioration in both structure and function underlie age-related cognitive decline. For example, the prefrontal cortex starts shrinking in the second decade of life, and the hippocampus begins shrinking in the sixth decade. Both of these areas exert key control over fluid intelligence (Raz et al., 2005). Improved understanding of the physiological factors, which may contribute to the maintenance of cognitive function integrity or serve as useful biomarkers to guide efforts toward the prevention of age-related cognitive decline, represents an additional priority research area.

Brain-derived neurotrophic factor (BDNF) is a neurotrophin that is widely distributed in the brain. BDNF and its tropomyosin-related receptor kinase B (TrkB) are highly expressed in cortical structures, especially in the hippocampus, and play an important role in learning and memory (Bekinschtein et al., 2008). BDNF is also expressed in muscle satellite cells (Mousavi and Jasmin, 2006) and can cross the blood-brain barrier in both directions (Poduslo and Curran, 1996; Pan et al., 1998), which supports the notion that circulating BDNF levels reflect BDNF expression in the brain (Ahlskog, 2011) and muscle satellite cells (Mousavi and Jasmin, 2006). BDNF is important for neurogenesis, phenotypic differentiation, and neuronal survival (Duman, 2002). In this context, research efforts have sought to assess the value of serum BDNF levels, which are correlated with BDNF expression in the brain (Huang et al., 2019), and improve our understanding of age-related cognitive decline. As a result, some studies, although not all, have associated serum BDNF levels with cognitive function-related outcomes (Kim et al., 2017; Ng et al., 2019). For example, lower serum BDNF concentrations have been associated with an increased risk of incident dementia in subsets of older adults (Weinstein et al., 2014; Ng et al., 2019), and evidence from intervention studies have provided support for the hypothesis that aerobic exercise has a beneficial impact on executive function and may be mediated by an exercise-related increase in BNDF (Kramer et al., 1999; Erickson et al., 2012; de Assis and de Almondes, 2017).

Reflecting the growing interest in the assessment of BDNF as a clinically useful biomarker for the prevention of cognitive decline, there has been an increasing number of observational studies reporting data on the association of circulating BDNF levels with cognitive functioning. Most studies to date, however, have been performed on patients with specific neuropsychiatric disorders and chronic diseases (Williams et al., 2017; Du et al., 2019; Jiang et al., 2019), and data from prospective studies associating circulating BDNF with a subsequent decline in overall or subdomain-specific cognitive decline in the broad community-dwelling older population remain scarce. In this context, and in line with the ultimate hypothesis that higher serum levels of BDNF at earlier stages of the cognitive decline process might reflect a beneficial compensatory response to pathophysiologic insults (Weinstein et al., 2014; Ng et al., 2019), we assessed whether higher serum BDNF levels were associated with lower risk of cognitive decline 2 years later in community-dwelling older adults free of dementia.

## Materials and Methods

### Study Participants

This report uses data from a subset of participants in the Otassha Study, a large observational study, of older adults living in areas surrounding the Tokyo Metropolitan Institute of Gerontology (TMIG), Tokyo, Japan. Study invitations were sent by postal mail to 6,699 community-dwelling older adults living in nine regions across the Itabashi Ward, in the Northwestern part of Tokyo. Nursing home residents were not eligible. A total of 876 study volunteers aged 65–84 years old were recruited for the study’s comprehensive baseline health assessment, which took place at a research TMIG research examination room in October 2011. Of those, a follow-up assessment was conducted for 410 participants in 2013. Included in this study were those who participated in both baseline and follow-up assessments, were free of a dementia diagnosis, and had data on serum BDNF samples and relevant cognitive tests (n = 405). All subjects provided written informed consent to participate in the study, which was approved by the Institutional Review Board and Ethics Committee of the TMIG (Acceptance No. 5, 1, 2011).

### Serum BDNF Measurements

Blood was drawn from participants within minutes, and samples were centrifuged at 3,000 rpm at 4°C for 15 min. Sera were then transferred to a new set of polyethylene tubes. The serum samples were stored at −80°C immediately after separate serum and serum BDNF levels were measured within 3 months using the BDNF Emax immunoassay system (Promega Corp., Madison, WI, United States), according to the supplier’s protocol (Promega, 2009). The sensitivity and specificity of the BDNF Emax ImmunoAssay System is 15.6 pg/mL of BDNF, and the cross-reactivity with other neurotrophic factors (NGF, NT-3, and NT-4) at 100 ng/mL being less than 3%. All samples were assayed in duplicate. The BDNF Emax ELISA kit used in this study likely reflects BDNF precursor (pro-BDNF) and BDNF mature form (Yoshida et al., 2012; Polacchini et al., 2015), the cross-reactivity is not specified (Yoshida et al., 2012; Polacchini et al., 2015). Cross-reactivity with other growth factors, such as neurogrowth factor, NT-3, and NT-4/5, is very low; i.e., less than 3% (Promega, 2009).

### Cognitive Function

Trained research assistants administered the Montreal Cognitive Assessment-Japanese version (MoCA-J) (Nasreddine et al., 2005; Fujiwara et al., 2010). For this study, we selected the MoCA-J as our outcome cognitive test for its increased sensitivity in the detection of mild cognitive impairment (MCI) (Petersen et al., 1999) in our community-dwelling study population, as compared to the MMSE. The MoCA-J is widely used and assesses several cognitive domains.

Details of the MoCA-J items have been described elsewhere (Fujiwara et al., 2010). Regarding the adaptation of the Japanese version of the MoCA scale, language cognition involves a naming task with low-familiarity animals (3 points), and the repetition of two complex sentences (2 points) in which attention is paid to whether or not participants maintain an equivalent level of syntax complexity in their answers using plain Japanese (Nasreddine et al., 2005). The full MoCA-J scale has scores range from 0 (worst) to 30 (best), while the 6 subscales have a narrower range, with lower and higher scores representing worse and better cognition, respectively: executive function (range 0–4), attention and working memory (range 0–6), delayed recall memory (range 0–5), language (range 0–5), orientation (range 0–6), and visuospatial (range 0–4). One point was added to the MoCA-J score if a participant had 12 years or less of formal education (Nasreddine et al., 2005; Fujiwara et al., 2010).

The outcome of interest was a meaningful short-term decline in MoCA-J performance. However, there are currently no well-established criteria to define what constitutes a meaningful decline in the full MoCA-J scale or its subscales. In this context, we arbitrarily used the following operational definition approach. First, for each participant, we calculated the difference in score between the participant’s baseline and follow-up assessments. Second, we examined the distribution of the within-person difference in scores and, for each cognitive outcome, identified the cut-off point that identified 25% of participants who declined the most in each cognitive outcome. For example, the subset of 25% of study participants who experienced the greatest decline in the full MoCA-J scale had a score decline of two or more points; thus, cognitive decline in the full MoCA-J scale was operationally defined as a decline in a score of two or more points in that scale. Analogously, for all MoCA-J subscales, cognitive decline was defined as a decline of one or more points from baseline to the follow-up visit 2 years later.

### Baseline Covariates

The following parameters collected at baseline were selected *a priori* from available data collected as part of available health assessment data as potential confounders: (a) socio-demographic variables, including age (continuous), sex (dichotomous), years of formal education (≥ vs. <12 years); (b) health habits, including smoking and drinking status (current vs. former vs. never for each health habit); and (c) clinical indicators, including a previous history of being told by a physician they had a diagnosis of hypertension, cardiac disease, diabetes, and/or stroke (≥ vs. < 2 diseases), and depressive symptoms. The latter was assessed using the Self-Rating Depression Scale (SDS) (Zung, 1965). Traditionally, SDS scores ≥ 50 have been suggested as indicative of a high depressive symptom burden [Dunstan et al., 2017; World Health Organization (WHO), 2021]; however, in community-dwelling older Japanese adults, a low cut-off score ≥ 40 has been proposed as a marker of greater depression symptoms burden (Ueno et al., 1997). The selection of covariates for potential inclusion in regression models was restricted in light of the sample size limitations.

### Statistical Analyses

Univariate distributions of study variables were reported as means ± standard deviations (SD), medians (interquartile range [IQR]), and proportions with absolute cell numbers. A bivariate analysis of baseline variables involved comparisons of the distribution of mean age, median cognitive scores, and categorical variables across the 3-level serum BDNF categorical variable using one-way analysis of variance, Kruskal-Wallis, and chi-square tests, respectively.

We also compared the observed proportion of study participants who met criteria for cognitive decline in each cognitive test score across categories of serum BDNF levels at baseline using chi-square tests for homogeneity of proportions. Multivariate logistic regression estimated the odds of meeting criterion for subsequent cognitive decline in the full MoCA-J scale as well as in its different subscales between baseline and follow-up visits as a function of serum BDNF levels at baseline. For the full MoCA-J scale, but not for the subscales, there was evidence that the association of baseline BDNF with subsequent cognitive decline differed between the subset of participants with better (full MoCA-J scale scores in the top three quartiles) vs. worse (full MoCA-J scale scores in the bottom quartile) at baseline. Specifically, the addition of the interaction term representing the multiplication of baseline full MoCA-J performance category (better vs. worse) times the 3-level serum BDNF levels at baseline resulted in a statistically significant improvement in model fit (*p* = 0.007, as per the likelihood ratio test). Thus, logistic regression models assessing the odds of cognitive decline the full MoCA-J scale was stratified by MoCA-J baseline performance.

Adjustments were conducted in a hierarchical fashion and similarly for all cognitive outcomes. For each MoCA-J-related outcome, three models were constructed: model 1 was unadjusted; model included adjustments for age (continuous), sex (dichotomous), and education (≥ vs. < 12 years); and model 3 included adjustments for all variables in model 2 plus a number of chronic diseases (2–4 vs. 0–1), depression symptom burden (Self-Rating Depression Scale scores ≥ vs. < 50), and baseline cognitive score. All above-listed covariates considered for adjustment were selected *a priori*. We also considered potential complementary adjustment for indicators of health habits (smoking status, and drinking habit). However, considering the small study sample size, and the lack of an association of those additional covariates considered for adjustment with baseline serum BDNF, they ended up not being included in final logistic regression models. As a post-hoc sensitivity analysis, we performed a re-analysis, excluding study participants who had a history of prevalent stroke at baseline. Stata version 14.2 software was used to this end (Stata Corporation, College Station, TX, United States).

## Results

Table 1 represents the distribution of selected characteristics of study participants at baseline. The group studied, which ranged in age from 65- to 84-years-old (mean age of 73.4 ± 4.9), consisted mainly of women (approximately 60%), and was highly educated (75% had 12 or more years of education). As per study inclusion criteria, none of the participants had been told previously by a physician that they had dementia. Less than 10% of study participants were current smokers, and less than 50% were current drinkers. Approximately one quarter of participants had Self-Rating Depression scale scores consistent with a high depression burden (SDS score ≥ 40) (Ueno et al., 1997). The average full MoCA-J score was slightly above that observed in a study that reported normative data in community-dwelling Japanese older adults (Narazaki et al., 2013). Distributions of characteristics across the bottom, intermediate, and top quartiles of BDNF are also shown in Table 1 as part of descriptive exploratory analyses; no statistically significant associations were observed.

**TABLE 1:** Distributions of characteristics of study participants across bottom, intermediate, and top quartiles of BDNF at baseline.

		Total sample		BDNF (ng/mL)						p*
				<6.9		6.9–11.7		> 11.7		
					
	n	405		103		199		103		
Age (years)	Mean ± SD	73.4	± 0.0	73.7	± 4.5	73.6	± 4.8	72.8	± 5.2	0.328
Female gender	n,%	241	59.5%	61	59.2%	123	61.8%	57	59.5%	0.553
Years of education	Median, [IQR]	12	[12–14]	12	[11–16]	12	[11–14]	12	[12–14]	0.614
	n, % of ≤ 12 years	257	63.6%	63	61.2%	127	63.8%	67	65.7%	
Full scale of MoCA-J	Median, [IQR]	24	[21–26]	24	[21–26]	24	[21–26]	24	[22–27]	0.703
[Range :0–30]	n, % of full scores	5	1.2%	1	1.0%	4	2.0%	0	0.0%	
Subscales of MoCA-J										
Executive Function	Median, [IQR]	3	[2–3]	3	[2–3]	3	[3–3]	3	[2–3]	0.686
[Range :0–4]	n, % of full scores	77	19.0%	14	13.6%	45	22.6%	18	17.5%	
Attention and Working Memory	Median, [IQR]	5	[4–6]	5	[5–6]	5	[4–6]	5	[4–6]	0.290
[Range :0–6]	n, % of full scores	161	39.8%	38	36.9%	80	40.2%	43	41.7%	
Language	Median, [IQR]	4	[4–5]	4	[3–5]	4	[4–5]	4	[4–5]	0.791
[Range :0–6]	n, % of full scores	35	8.6%	7	6.8%	18	9.0%	10	9.7%	
Orientation	Median, [IQR]	6	[6–6]	6	[6–6]	6	[6–6]	6	[6–6]	0.916
[Range :0–6]	n, % of full scores	344	84.9%	86	83.5%	171	85.9%	87	84.5%	
Delayed Recall	Median, [IQR]	3	[1–4]	2	[0–4]	3	[1–4]	3	[1–4]	0.812
[Range :0–5]	n, % of full scores	44	10.9%	11	10.7%	19	9.5%	14	13.6%	
Visuospatial	Median, [IQR]	4	[3–4]	4	[3–4]	4	[3–4]	4	[3–4]	0.424
[Range :0–4]	n, % of full scores	264	65.2%	73	70.9%	127	63.8%	64	62.1%	
Smoking status										
Current	n,%	35	8.7%	5	4.9%	18	9.0%	12	11.8%	0.503
Past	n,%	115	28.5%	29	28.2%	57	28.6%	29	28.4%	
Never	n,%	254	62.9%	69	67.0%	124	62.3%	61	59.8%	
Drinking status										
Current	n,%	199	49.1%	55	53.4%	100	50.3%	44	42.7%	0.987
Past	n,%	37	9.1%	8	7.8%	12	6.0%	17	16.5%	
Never	n,%	169	41.7%	40	38.8%	87	43.7%	42	40.8%	
Chronic diseases										
Hypertension	n,%	163	40.2%	34	33.0%	87	43.7%	42	40.8%	0.197
Heart diseases	n,%	61	15.1%	23	22.3%	25	12.6%	13	12.6%	0.058
Diabetes	n,%	51	12.6%	10	9.7%	28	14.1%	13	12.6%	0.343
Stroke	n,%	27	6.7%	4	3.9%	17	8.5%	6	5.8%	0.283
Number Chronic Diseases										
0	n,%	185	45.7%	56	54.4%	85	42.7%	44	42.7%	0.166
1	n,%	149	36.8%	27	26.2%	77	38.7%	45	43.7%	
2	n,%	60	14.8%	16	15.5%	31	15.6%	13	12.6%	
≥ 3	n,%	11	2.7%	4	3.9%	6	3.0%	1	1.0%	
Depressive symptoms assessed by Self-Rating Depression Scale	n,% of ≥ 40	97	24.0%	18	17.5%	52	26.1%	27	26.2%	0.204

BDNF, brain-derived neurotrophic factor; MoCA-J, the Montreal Cognitive Assessment-Japanese version. *Comparisons of the distribution of mean age, median cognitive scores, and categorical variables across the 3-level serum BDNF categorical variable were conducted using, one-way ANOVA, Kruskal-Wallis, and chi-square tests, respectively.

**FIGURE 1:**
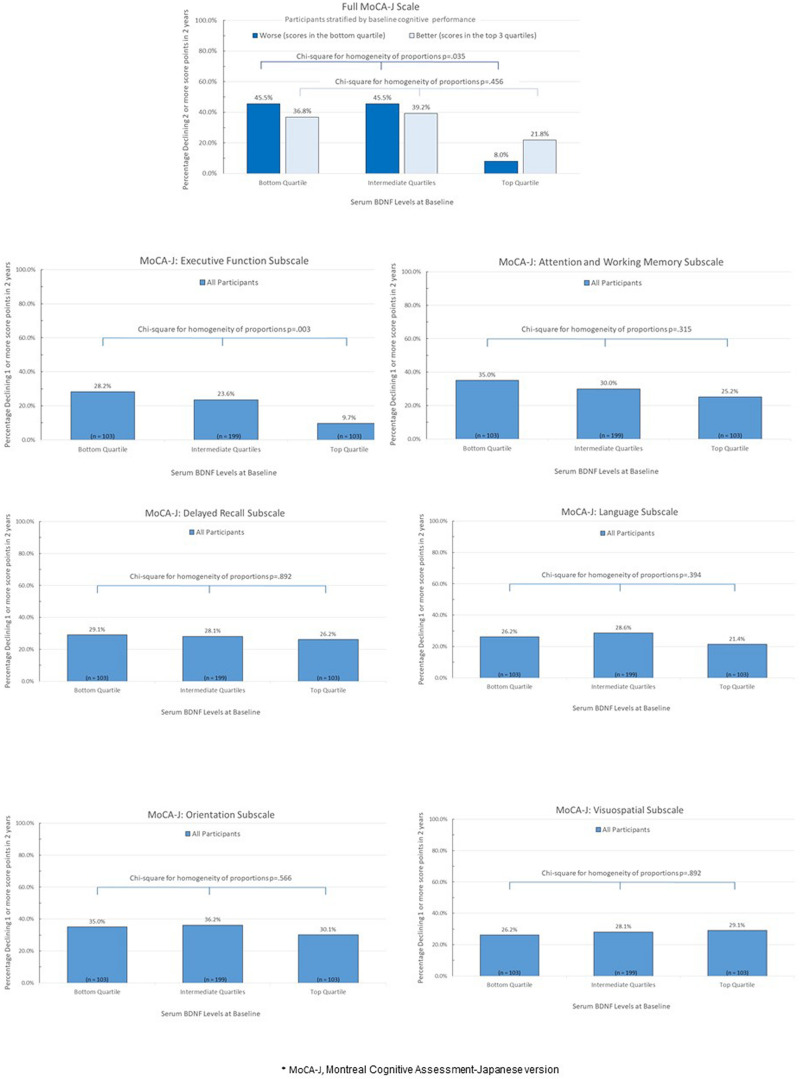
Percentage of study paticipants experiencing cognitive decline from baseline to follow-up visit 2 years later according to quartiles of serum BDNF at baseline*.

Figure 1 represents crude proportions of study participants meeting criterion for cognitive decline in the full MoCA-J and its subscales from baseline to follow-up visit 2 years later according to quartiles of serum BDNF at baseline. The proportion who experienced a cognitive decline in the full MoCA-J scale was substantially lower in those with the highest (i.e., top quartile) BDNF levels at baseline, but statistical significance was only reached in the stratum that included participants whose full MoCA-J scores at baseline were in the lowest quartile (i.e., worst performance, *p* = 0.035). Dose-response relationships characterized by incrementally higher baseline BDNF levels with an incrementally lower proportion of participants experiencing cognitive decline were observed in the executive function subscale, as well as in the attention and working memory subscale, though the dose-response relationship was stronger and only statistically significant for the executive function subscale (*p* = 0.003).

Logistic regression models estimated adjusted odds of cognitive decline between baseline and follow-up visit 2 years later as a function of baseline levels of BDNF (bottom vs. intermediate vs. top quartiles), adjusted for age, sex, education, number of chronic diseases, depressive symptom burden, and cognitive score at baseline. Among participants with baseline full MoCA-J scale scores ≤ 21 (worst quartile), those with the highest baseline BDNF levels were associated with substantially lower odds of cognitive decline in the full MoCA-J scale from baseline to the follow-up visit, as compared those with lowest BDNF levels; i.e., OR: 0.10 (95% CI: 0.02–0.62; *p*-value = 0.013). There was no association between serum BDNF levels at baseline and subsequent decline among those who performed better (scores in the top three quartiles) in the full MoCA-J scale at baseline. Regarding the MoCA-J executive function subscale, the odds of cognitive decline from baseline to the follow-up visit was substantially lower among those with highest than lowest baseline BDNF levels; i.e., OR: 0.27 (95% CI: 0.13–0.60; *p*-value = 0.001). No other meaningful, statistically significant association of serum BDNF levels at baseline with other MoCA-J subscales. Post-hoc analyses similar to those presented in Table 2 were repeated after excluding those who had a history of stroke at baseline, yielding analogous results (data not shown).

**TABLE 2:** Multiple logistic regression estimating the odds of subsequent cognitive decline between baseline and follow-up visits as a function of quartiles of serum BDNF at baseline*.

			Model 1**		Model 2**		Model 3**	
Cognitive decline		Baseline BDNF levels	OR (95%CI)	*p*-value	OR (95%CI)	*p*-value	OR (95%CI)	*p*-value
**Full MOCA-J Scale**								
	Those whose baseline full MOCA-J scale scores were in the bottom quartile (worse)	Lower (Q1)	1 (reference)		1 (reference)		1 (reference)	
		Middle (Q2-3)	0.41 (0.14 – 1.20)	0.098	0.34 (0.11 – 1.10)	0.063	0.39 (0.12 – 1.20)	0.111
		Higher (Q4)	0.15 (0.03 –0.76)	0.023	0.11 (0.02 –0.62)	0.013	0.10 (0.02 –0.62)	0.013
	Those whose baseline full MOCA-J scale scores were in the top 3 quartiles (better)	Lower (Q1)	1 (reference)		1 (reference)		1 (reference)	
		Middle (Q2-3)	1.10 (0.62 – 2.00)	0.732	1.20 (0.64 – 2.20)	0.594	1.10 (0.62 – 2.10)	0.668
		Higher (Q4)	0.76 (0.39 – 1.50)	0.426	0.99 (0.49 – 2.00)	0.970	0.93 (0.45 – 1.90)	0.836
**MOCA-J Subscales**								
	Executive function	Lower (Q1)	1 (reference)		1 (reference)		1 (reference)	
		Middle (Q2-3)	0.74 (0.41 – 1.30)	0.298	0.79 (0.46 – 1.4)	0.404	0.79 (0.46 – 1.40)	0.390
		Higher (Q4)	0.23 (0.10 –0.52)	< 0.001	0.28 (0.13 –0.61)	0.001	0.27 (0.13 –0.60)	0.001
	Attention and working memory	Lower (Q1)	1 (reference)		1 (reference)		1 (reference)	
		Middle (Q2-3)	0.80 (0.48 – 1.30)	0.377	0.81 (0.48 – 1.30)	0.408	0.80 (0.47 – 1.40)	0.406
		Higher (Q4)	0.63 (0.34 – 1.10)	0.130	0.64 (0.35 – 1.20)	0.155	0.63 (0.34 – 1.20)	0.159
	Delayed recall	Lower (Q1)	1 (reference)		1 (reference)		1 (reference)	
		Middle (Q2-3)	0.95 (0.56 – 1.60)	0.857	0.95 (0.56 – 1.60)	0.854	0.92 (0.51 – 1.70)	0.786
		Higher (Q4)	0.86 (0.47 – 1.60)	0.640	0.90 (0.49 – 1.70)	0.748	0.75 (0.38 – 1.50)	0.409
	Language	Lower (Q1)	1 (reference)		1 (reference)		1 (reference)	
		Middle (Q2-3)	1.10 (0.66 – 1.90)	0.655	1.10 (0.66 – 1.90)	0.670	1.00 (.56 – 1.80)	0.983
		Higher (Q4)	0.76 (0.40 – 1.50)	0.414	0.80 (0.42 – 1.50)	0.503	0.70 (0.35 – 1.40)	0.316
	Orientation	Lower (Q1)	1 (reference)		1 (reference)		1 (reference)	
		Middle (Q2-3)	1.10 (0.64 – 1.70)	0.833	1.10 (0.64 – 1.80)	0.809	1.00 (0.62 – 1.70)	0.879
		Higher (Q4)	0.80 (0.45 – 1.40)	0.457	0.82 (0.46 – 1.50)	0.516	0.70 (0.44 – 1.50)	0.487
	Visuospatial	Lower (Q1)	1 (reference)		1 (reference)		1 (reference)	
		Middle (Q2-3)	1.10 (0.64 – 1.90)	0.731	1.10 (0.64 – 1.90)	0.731	1.30 (0.70 – 2.20)	0.441
		Higher (Q4)	1.20 (0.66 – 2.10)	0.640	1.20 (0.66 – 2.30)	0.516	1.50 (0.77 – 2.90)	0.230

*BDNF, Brain-derived neurotrophic factor; Q, quartile; OR, odds ratio; CI, confidence interval. **Model 1: unadjusted; Model 2: adjusted for baseline age, gender, and education; and Model 3: adjusted for baseline age, gender, education, baseline cognitive score, number of chronic diseases, and depressive symptoms burden.

## Discussion

This observational, prospective study documented associations between higher serum BDNF levels with decreased odds of cognitive decline 2 years later in community-dwelling older adults. Higher serum BDNF levels were independently associated with substantially lower odds of decline in executive function in the total study population, and decline in the full MoCA-J scale in the subset of participants with lower baseline scores ≤ 21 (bottom quartile) in the full MoCA-J scale, consistent with a mild cognitive impairment status (Narazaki et al., 2013; Carson et al., 2018).

We studied a community-dwelling population that had a high education level and did not include subjects with a known clinical diagnosis of dementia. The average full MoCA-J scale score was slightly above that observed in a population-based sample used for the derivation of normative data on the distribution of MoCA-J scores in a community-dwelling older Japanese population. Our findings are thus consistent with the hypothesis that higher serum levels of BDNF at earlier stages of the cognitive decline process might reflect an effective compensatory response to pathophysiologic insults (Laske et al., 2006; Weinstein et al., 2014; Ng et al., 2019). This stands in contrast to the lower serum BDNF levels observed in patients with advanced dementia stages, possibly due to compromised compensatory capacity (Weinstein et al., 2014; Ng et al., 2019).

Our observational data suggest that the relationship between serum BDNF levels and cognition in community-dwelling older populations might vary by cognitive subdomain. We found a relationship between higher serum BDNF and executive function in the whole population, but not in other MoCA-J subscales reflective of other cognitive subdomains. The reason underlying these differential associations by cognitive subdomains remains unclear. Considering previous evidence that decline in executive function, which involves higher-order cognitive abilities, often precedes a decline in other cognitive subdomains (Carlson et al., 2009), it might be possible that the impact of lower serum BDNF levels on cognitive function could, in community-dwelling older populations with overall cognitive status relatively preserved, be first noticeable in executive function, and, subsequently, in other domains. The small sample size and the short-term follow-up of our study, along with the current lack of prospective data in the literature addressing differential relationships of serum BDNF levels with diverse cognitive subdomains prevents stronger speculations regarding the possible cognitive domain-specific effects associated with serum BDNF levels in community-dwelling older populations.

Our results should be interpreted in light of important study limitations. The follow-up period was short (2 years), and the sample size relatively small. There is no validated, widely accepted standard for defining what constitutes clinically meaningful score declines in the full MoCA-J or its subscales; thus, we used an arbitrary operational definition for cognitive decline in study analyses. The cohort solely consisted of Japanese adults living in an urban area of Tokyo; therefore, the influence of race and lifestyle was limited. This was an observational study, which restricts inferences on causality, or the potential impact of intervention-resulting changes in serum BDNF levels on cognition. Cognitive decline was defined using a statistical distribution-based operational definition, given the lack of an established standard for what constitutes clinically meaningful score declines in the full MoCA-J and its subscales. This study’s strengths include its population-based setting, data collection done in a standardized fashion, and the contribution of novel data on the prospective relationship of serum BDNF levels as a biomarker for declines in different cognitive subdomains in community-dwelling older adults.

### Conclusion and Implications

Our results show that higher serum BDNF levels were associated with a lower risk of decline in cognitive function in community-dwelling older adults. They also demonstrate that the type of cognitive decline and the subsets of individuals at risk may vary according to cognitive subdomains and baseline cognitive performance level. This study contributes information relevant for public health researchers seeking to establish the value of serum BDNF levels as part of novel interventions and approaches to prevent age-related cognitive decline.

## Data Availability Statement

The raw data supporting the conclusions of this article will be made available by the authors, without undue reservation.

## Ethics Statement

The studies involving human participants were reviewed and approved by the Institutional Review Board and Ethic Committee of the TMIG. The patients/participants provided their written informed consent to participate in this study. Written informed consent was obtained from the individual(s) for the publication of any potentially identifiable images or data included in this article.

## Author Contributions

YF, MHc, and KI: conceptualization. HK: data curation. YF and MHc: formal analysis. YF, MHc, HS, MHs, HK, HH, SO, MK, and KI: investigation. HK, HH, HS, YF, SO, and KI: project administration. SO and KI: resources. YF: writing–original draft. YF, MHc, PC, and KI: writing–review and editing. All authors have read and agreed to the published version of the manuscript.

## Conflict of Interest

The authors declare that the research was conducted in the absence of any commercial or financial relationships that could be construed as a potential conflict of interest.
